# Angiotensin-(1–7) attenuates organ injury and mortality in rats with polymicrobial sepsis

**DOI:** 10.1186/s13054-018-2210-y

**Published:** 2018-10-27

**Authors:** Hsin-Jung Tsai, Mei-Hui Liao, Chih-Chin Shih, Shuk-Man Ka, Cheng-Ming Tsao, Chin-Chen Wu

**Affiliations:** 1 Department of Anesthesiology, Taipei Veterans General Hospital and National Yang-Ming University, No. 201, Sec. 2, Shipai Road, Beitou District, Taipei, 11217 Taiwan, Republic of China; 2 Department of Pharmacology, National Defense Medical Center, Taipei, Taiwan, Republic of China; 3 Graduate Institute of Aerospace and Undersea Medicine, National Defense Medical Center, Taipei, Taiwan, Republic of China; 4 Department of Anesthesiology, Tri-Service General Hospital, National Defense Medical Center, Taipei, Taiwan, Republic of China

**Keywords:** Angiotensin-(1–7), Apoptosis, Inflammatory response, Organ injury, Polymicrobial sepsis

## Abstract

**Background:**

Sepsis and related multiple organ dysfunction result in high morbidity and mortality. Angiotensin (Ang)-(1–7), a biologically active peptide, has various opposing effects of Ang II. Because the effect of Ang-(1–7) on sepsis is unknown, in this study we aimed to determine the impact of Ang-(1–7) on pathophysiologic changes in a clinically relevant model of polymicrobial sepsis induced by cecal ligation and puncture (CLP).

**Methods:**

Sepsis was induced by CLP in rats under anesthesia. Rats were randomized to one of the following five groups: (1) sham-operated group, (2) Ang-(1–7) (1 mg/kg intravenously infused for 1 h) at 3 h and 6 h after sham operation, (3) CLP, (4) Ang-(1–7) at 3 h after CLP, and (5) Ang-(1–7) at 3 h and 6 h after CLP. Rats were observed for 24 h after CLP surgery and then killed for subsequent histological examination.

**Results:**

Ang-(1–7) significantly improved the survival of septic rats (83.3% vs. 36.4% at 24 h following CLP; *p* = 0.009). Ang-(1–7) attenuated the CLP-induced decreased arterial pressure and organ dysfunction, indicated by diminished biochemical variables and fewer histological changes. Ang-(1–7) significantly reduced the level of plasma interleukin-6 and pulmonary superoxide production (*p* < 0.05). Moreover, caspase-3 and cytoplasmic IκB expression in liver was significantly lower in the Ang-(1–7)-treated CLP rats (*p* < 0.05).

**Conclusions:**

In this clinically relevant model of sepsis, Ang-(1–7) ameliorates CLP-induced organ dysfunction and improves survival, possibly through suppressing the inflammatory response, oxidative stress, and apoptosis, suggesting that Ang-(1–7) could be a potential novel therapeutic approach to treatment of peritonitis and polymicrobial sepsis.

## Background

Sepsis remains the major cause of mortality and morbidity in intensive care [1, 2], despite significant advances in understanding of the biologic alterations and improvements in medical care. Sepsis is caused by the release of proinflammatory cytokines and ROS, as well as tissue cell apoptosis, leading to development of multiple organ injuries [3]. In addition, cross-talk between these processes has been recognized in sepsis [3]. However, no specific and effective pharmacological intervention for sepsis is currently available.

Angiotensin (Ang)-(1–7), a biologically active peptide of the renin-angiotensin system (RAS), is produced mainly through angiotensin-converting enzyme 2 (ACE2) using primarily angiotensin II (Ang II) as a substrate [4, 5]. Through binding its G protein-coupled receptor Mas [6], Ang-(1–7) counteracts various effects of Ang II, such as vasoconstriction, inflammation, proliferation, and apoptosis [7]. Therefore, the ACE2/Ang-(1–7)/Mas axis contributes to the regulation of vascular tone, as well as inflammatory responses and organ function after injury [5, 8].

Accumulating evidence indicates that ACE2 plays an important role in severe lung injury [9, 10], and it attenuates endotoxin-induced lung via the Ang-(1–7)/Mas pathway by inhibiting NF-κB activation [11]. It has been reported that Ang-(1–7) has its anti-inflammatory effects in arthritis and allergic airway through the Mas receptor [12, 13] and decreases lipopolysaccharide (LPS)-stimulated proinflammatory cytokines, including tumor necrosis factor-α and interleukin 6 (IL-6), in mouse peritoneal macrophages [14]. Moreover, Ang-(1–7) attenuates cytokine production, hypothermia, and mortality from LPS-induced endotoxemia; however, the underlying molecular mechanism remains elusive [15]. In addition, apoptosis plays a major role in the pathogenesis of sepsis [16], which is involved in sepsis-induced organ dysfunction [3]. Furthermore, Ang-(1–7)/Mas signaling could inhibit LPS-induced apoptosis in alveolar epithelial cells [17] and pulmonary microvascular endothelial cells [18].

A variety of animal species have been used to study septic responses induced by endotoxin. However, cecal ligation and puncture (CLP) is the most frequently used model of polymicrobial sepsis because it closely resembles the progression and characteristics of human sepsis [19]. Therefore, we applied a CLP approach to induce sepsis in rats in this study, and we investigated whether Ang-(1–7) exerts beneficial effects against CLP-induced organ injuries and death via suppressing inflammation and apoptosis pathways.

## Methods

We used male adult Wistar rats (280 to 320 g; BioLASCO Taiwan Co., Taipei, Taiwan) in this study. The animals were housed in 12-h/12-h light/dark conditions with free access to food and tap water. This study was approved by the local institutional review board, and the experiments were performed in compliance with the National Institutes of Health guidelines for the treatment of animals and for ethical animal research.

### Surgical procedures

Under anesthesia with intraperitoneal sodium pentobarbital (50 mg/kg), polyurethane catheters were inserted into the left carotid artery for blood pressure monitoring and blood sampling and into the right internal jugular vein for drug administration. Adequate anesthesia in animals was verified by pinching their toes. The catheters were exteriorized and fixed to the back of the neck. Subsequently, the cannulated rats were allowed to recover to their normal condition with standardized pellet food and tap water ad libitum.

On the next day, intraperitoneal sepsis was induced by CLP under anesthesia with 50 mg/kg intravenous pentobarbital [20]. Briefly, a laparotomy was made through a 2-cm-long midline incision in the lower abdomen. The cecum was exposed and ligated with a 3-0 silk ligature just distal to the ileocecal valve, punctured twice at opposite ends using an 18-gauge needle. The bowel was then returned into the abdominal cavity, and the incision was closed. In addition, 0.2% lidocaine was used to infiltrate the incision wound after abdominal closure for analgesia. The rats in the sham operation (SOP) group underwent similar laparotomy and cecal exposure without CLP. All animals were treated subcutaneously with 0.9% NaCl solution (10 ml/kg) immediately after laparotomy.

All rats enrolled in the study were kept in a small in-house animal facility to enable optimal monitoring, and their overall health status was checked every 3 h for signs of distress, such as shallow and rapid respiration, piloerection, and hunching or cowering in the corner of the cage. Rats were killed only 24 h following CLP or sham surgery or upon signs of imminent death (that is, unresponsive to external stimuli, inability to maintain an upright position, tremor, and prolonged/deep hypothermia and/or agonal breathing) via an overdose of pentobarbital (100 mg/kg intravenously). The survival rate during the study in each group was further analyzed. In regard to “blinding” implemented in this study, an investigator blinded to the treatment group made the assessment for survival. In addition, analysis of the pathologic examination and the statistical results were done in a blinded fashion.

### Experimental protocol

**Fig. 1:**
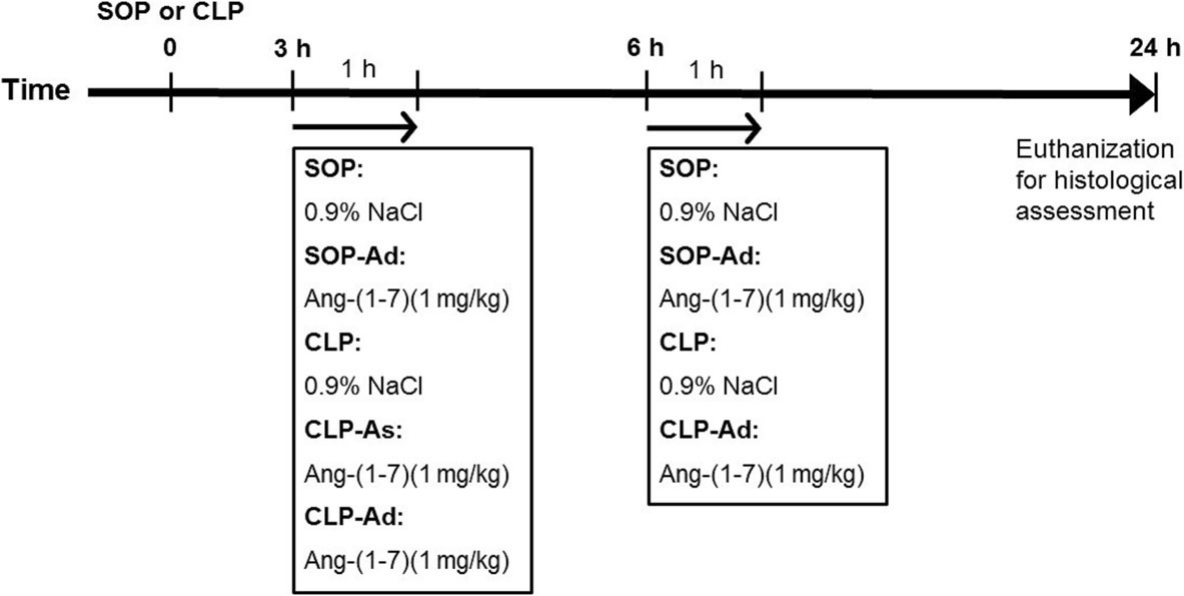
Experimental design. Timeline of 24-h experiments. SOP = sham operation, 0.9% NaCl = normal saline (intravenously for 1 h), Ang-(1–7) = angiotensin-(1–7) (1 mg/kg intravenously for 1 h), SOP-Ad = Ang-(1–7) at 3 and 6 h after sham operation, CLP-Ad = Ang-(1–7) at 3 and 6 h after CLP procedure, CLP-As = Ang-(1–7) at 3 h only after CLP procedure

Rats received CLP or SOP in randomized fashion by flipping a coin to one of five treatment groups (Fig. 1): (1) SOP group: an intravenous infusion of 0.9% NaCl for 1 h at 3 and 6 h after SOP (*n* = 6), (2) SOP-Ad group: an intravenous infusion of 1 mg/kg Ang-(1–7) for 1 h at 3 and 6 h after SOP (*n* = 6), (3) CLP group: same regimen and volume of saline at 3 and 6 h after CLP procedure (*n* = 8), (4) CLP-As group: same regimen of Ang-(1–7) at 3 h after CLP procedure (*n* = 3), or (5) CLP-Ad group: same regimen of Ang-(1–7) at 3 and 6 h after CLP procedure (*n* = 8). Ang-(1–7) (Tocris Bioscience, Bristol, UK) was dissolved in 0.9% NaCl, and its dosage was selected according to a previous study [15].

### Measurement of hemodynamic parameters

At 0, 3 and 24 h after CLP or SOP in all animals, mean arterial pressure (MAP) and heart rate (HR) were monitored by connecting the arterial catheter to a pressure transducer (P23ID; Statham, Oxnard, CA, USA) and displaced on a polygraph recorder (MacLab/4e; AD Instruments Pty Ltd., Castle Hill, Australia).

### Quantification of organ function

At 0, 3 and 24 h following CLP or sham surgery, 1 ml of arterial blood was collected, and 10 μl of the blood sample was analyzed for blood glucose by using a One-Touch II blood glucose monitoring system (Lifescan Inc., Milpitas, CA, USA). The remainder of the collected blood was then centrifuged (3 min, 16,000 *g*) and analyzed for plasma levels of alanine aminotransferase (ALT), blood urea nitrogen (BUN), creatinine, and lactate dehydrogenase (LDH) by Fuji DRICHEM 3030 (Fuji Photo Film Co., Ltd., Tokyo, Japan). Each volume of blood withdrawn was replaced immediately by the infusion of an equal volume of saline. Owing to blood clots or kinking of the arterial catheter, blood samples could not be obtained in some of the rats surviving the whole experimental period, especially in rats that underwent the CLP procedure.

### Measurement of plasma IL-6 concentrations

Collected plasma at 0 and 24 h after sham or CLP surgery was analyzed for IL-6 in duplicate by using enzyme-linked immunosorbent assay kits (R&D Systems, Inc., Minneapolis, MN, USA) according to the manufacturer’s instructions.

### Measurement of superoxide production

At the end of the experiment, the tissues of lung, liver, and kidney were removed and placed on the scintillation plates. These microplates containing Krebs-HEPES buffer (100 μl) and 1.25 mM lucigenin (50 μl) were placed into a microplate luminometer (Hidex Microplate Luminometer, Turku, Finland) for analysis of superoxide ion as previously described [20]. The obtained values were measured in duplicate, and all tissues were then dried in a laboratory oven for 24 h. The superoxide levels in all tissues were expressed as counts per second in each milligram of dry tissue.

### Western blot analysis

For cleaved caspase-3 and nuclear factor of kappa light polypeptide gene enhancer in B-cells inhibitor (IκB) expression analysis [11, 18, 21], supernatants of homogenized liver tissues (100 μg total protein) were separated by electrophoresis in a 10–12% polyacrylamide gel and transferred onto nitrocellulose membrane (Mini Trans-Blot Cell; Bio-Rad Laboratories, Hercules, CA, USA). The membranes were blocked with 5% albumin (BioShop Canada Inc., Burlington, ON, USA) in Tris-buffered solution containing 0.1% Tween-20 (TBST) for 1.5 h at room temperature and then incubated overnight at 4 °C with primary antibody (cleaved caspase 3, 1:500 dilution, Cell Signaling Technology Inc., Danvers, MA, USA; IκB, 1:5000 dilution, Abcam, Cambridge, UK) in TBST buffer, which was followed by the addition of a horseradish peroxidase-conjugated goat antirabbit immunoglobulin G (Cell Signaling Technology Inc.). The protein expression was visualized using Pierce enhanced chemiluminescence Western blotting reagent (Thermo Fisher Scientific, Rockford, IL, USA), followed by exposure to radiographic film. The densitometry of bands was quantified.

### Histological examination

Specimens of liver and lung were fixed in 10% formaldehyde for more than 24 h, which was followed by dehydration in graded ethanol. The tissues were embedded in paraffin wax, sectioned into 4-μm-thick slices, and stained with hematoxylin and eosin. The severity of lung injury (lung edema, pulmonary congestion, thickening of the alveolar wall, and areas of inflammatory infiltration) and liver necrosis was scored from 0 (none or minimal) to 3 (severe) by a pathologist in a blinded fashion in three animals from each group. The score of ten randomly selected high-power fields was used for assessment.

### Statistical analysis

Shapiro-Wilk tests were used to evaluate whether collected variables were normally distributed. Variables that did not follow the normal distribution were log-transformed. Analysis of variance for repeated measures was used to compare physiological parameters among groups. For the remaining parameters (including superoxide production and caspase-3 and IκB expression), the significance of differences was analyzed by one-way analysis of variance combined with the Newman-Keuls post hoc test. The severity score of tissue injury was compared between the CLP and CLP-Ad groups by the Mann-Whitney *U* test. Survival distribution was compared using the log-rank test and represented by Kaplan-Meier curves. Data are presented as median and IQR. A *p* value less than 0.05 was considered statistically significant. Regarding the power analysis of our findings, Schoenfeld’s formula for survival models was used to estimate post hoc power. Given a two-sided type I error rate of 0.05, a hazard ratio of 2, and our sample size of 12 and 22 in the CLP-Ad and CLP groups, respectively, a power level of more than 0.95 could be attained in the corresponding analyses.

## Results

### Survival

**Fig. 2:**
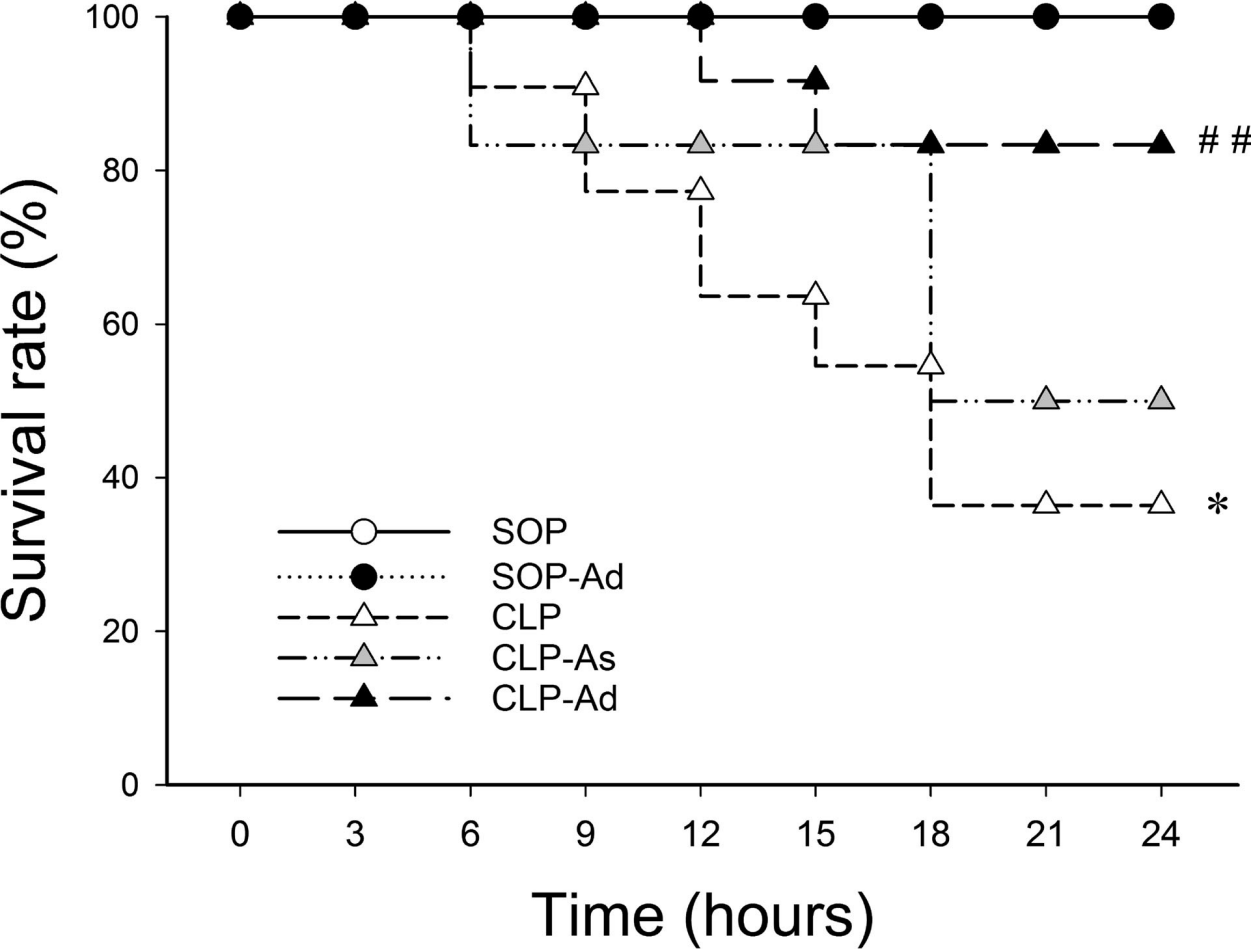
Angiotensin-(1–7) improves survival after polymicrobial sepsis. Analysis of the survival rate after the administration of angiotensin-(1–7) to rats that underwent sham operation (SOP, *n* = 6), SOP plus angiotensin-(1–7) administration (1 mg/kg at 3 and 6 h after SOP procedure [SOP-Ad], *n* = 6), cecal ligation and puncture (CLP, *n* = 22), CLP plus angiotensin-(1–7) administration (1 mg/kg at 3 h after CLP procedure [CLP-As], *n* = 6), and CLP plus angiotensin-(1–7) administration (1 mg/kg at 3 and 6 h after CLP procedure [CLP-Ad], *n* = 12). Survival time was measured for 24 h. Survival distribution was compared using the log-rank test and represented by Kaplan-Meier curves. Data are expressed as percentage of rats that survived at the end of the experiment. *p < 0.05 CLP. vs. SOP. ^##^*p* < 0.01 CLP-Ad vs. CLP

Comparing the overall survival, there was a significant difference among the five study groups (*p* = 0.002). In the CLP group, 2 of 22 animals died at 9 h (survival: 20 of 22 rats, 90.9%), and 14 of 22 rats died at 24 h after CLP procedure (survival: 8 of 22 rats, 36.4%), whereas no mortality was observed within 24 h in all SOP groups (survival: 6 of 6 rats, 100% in each SOP group) (Fig. 2). In the CLP-As group, one of six rats died at 9 h (survival: five of six rats, 83.3%), and three of six rats died at 24 h after CLP procedure (survival: three of six rats, 50%). In the CLP-Ad group, however, no mortality was observed at 9 h (survival: 12 of 12 rats, 100%), and only 2 of 12 rats died at 24 h after CLP procedure (survival: 10 of 12 rats, 83.3%). Thus, survival was higher in CLP rats with double-dose Ang-(1–7) treatment (*p* = 0.009 vs. CLP group).

### Cardiovascular changes

**Fig. 3:**
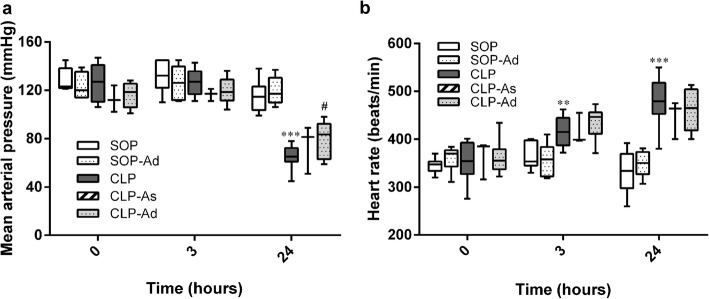
Effects of angiotensin-(1–7) on changes in hemodynamic parameters in rats that underwent cecal ligation and puncture. Depicted are the changes in (**a**) mean arterial blood pressure and (**b**) heart rate during the experimental period. Rats underwent sham operation (SOP, *n* = 6), SOP plus angiotensin-(1–7) administration (1 mg/kg at 3 and 6 h after SOP procedure [SOP-Ad], *n* = 6), cecal ligation and puncture (CLP, *n* = 8), CLP plus angiotensin-(1–7) administration (1 mg/kg at 3 h after CLP procedure [CLP-As], *n* = 3), and CLP plus angiotensin-(1–7) administration (1 mg/kg at 3 and 6 h after CLP procedure [CLP-Ad], *n* = 8). Analysis of variance for repeated measures was used for analysis of time × group interaction. Data are shown as median and IQR. **p < 0.01, ***p < 0.001 CLP. vs. SOP; ^#^*p* < 0.05 CLP-As or CLP-Ad vs. CLP

As shown in Fig. 3, baseline data of MAP and HR did not significantly differ among groups. After the CLP procedure, MAP was significantly decreased at 24 h (*p* < 0.001 vs. SOP group) (Fig. 3a). This CLP-induced severe hypotension was significantly attenuated by double-dose Ang-(1–7) (*p* = 0.037 vs. CLP group) (Fig. 3a), but not by single-dose Ang-(1–7). Meanwhile, CLP led to a significant and sustained increase in HR from 3 to 24 h (*p* < 0.01 vs. SOP group) (Fig. 3b), which was not attenuated in rats treated with Ang-(1–7) (*p* = 0.42 vs. CLP group) (Fig. 3b). In addition, MAP and HR in Ang-(1–7)-treated SOP rats did not significantly differ from the SOP rats treated with saline during the experimental period.

### Plasma indexes of organ injury and blood glucose

**Fig. 4:**
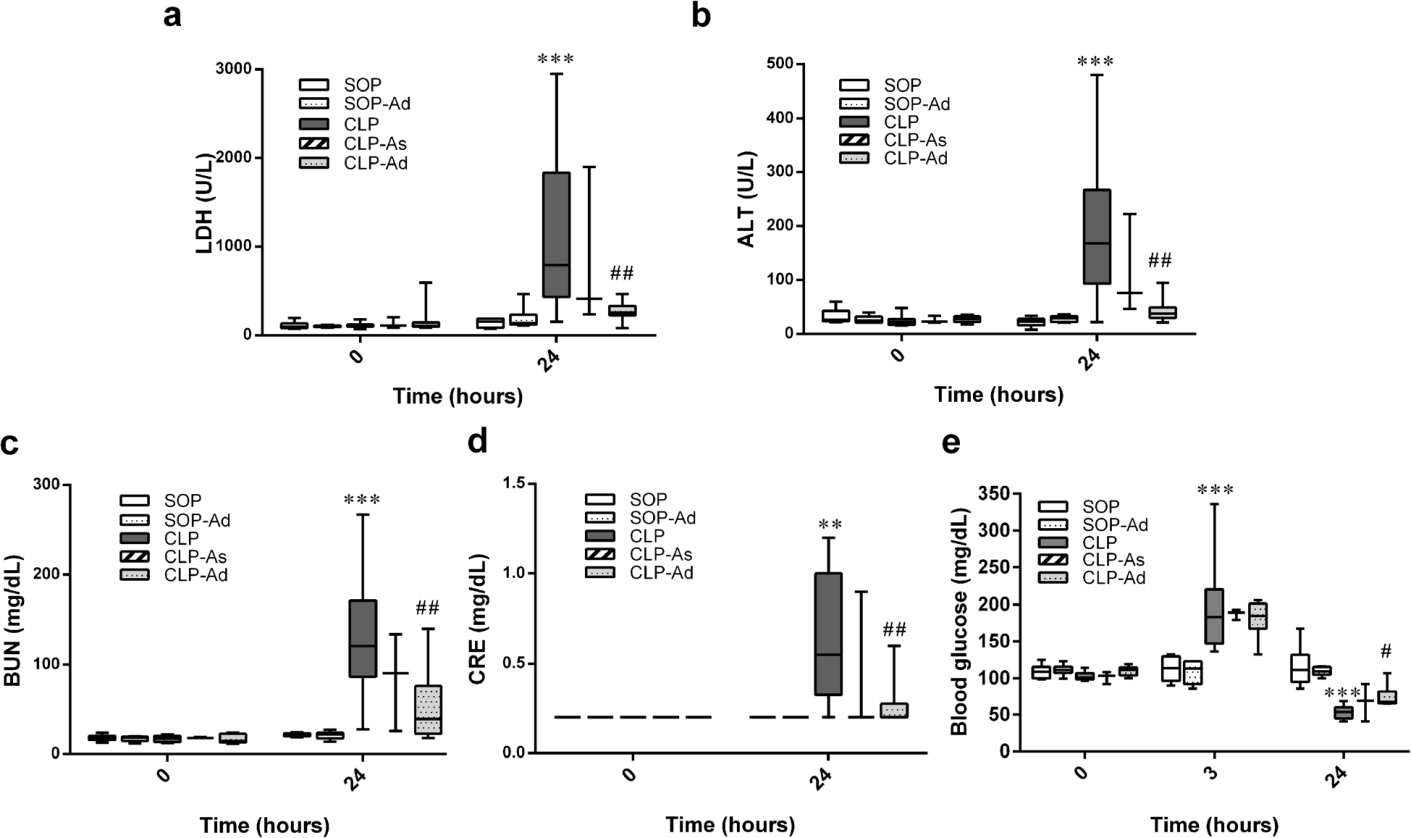
Effects of angiotensin-(1–7) on biochemical parameters in rats that underwent cecal ligation and puncture. These parameters include (**a**) lactate dehydrogenase, (**b**) alanine aminotransferase (ALT), (**c**) blood urea nitrogen, (**d**) creatinine (CRE), and (**e**) blood glucose during the experimental period. Rats underwent sham operation (SOP, *n* = 6), SOP plus angiotensin-(1–7) administration (1 mg/kg at 3 and 6 h after SOP procedure [SOP-Ad], *n* = 6), cecal ligation and puncture (CLP, *n* = 8), CLP plus angiotensin-(1–7) administration (1 mg/kg at 3 h after CLP procedure [CLP-As], *n* = 3), and CLP plus angiotensin-(1–7) administration (1 mg/kg at 3 and 6 h after CLP procedure [CLP-Ad], *n* = 8). Owing to blood clots or kinking of the arterial catheter, blood samples could not be obtained in some of rats surviving the whole experimental period. Analysis of variance for repeated measures was used for analysis of time × group interaction. Data are shown as median and IQR. **p < 0.01, ***p < 0.001 CLP. vs. SOP; ^#^*p* < 0.05, ^##^*p* < 0.01 CLP + As or CLP + Ad vs. CLP

We log-transformed for the biochemical markers, including LDH, ALT, BUN, and creatinine, to normalize these data and for statistical analysis. Baseline biochemical data did not significantly differ among all groups (Fig. 4). In the SOP and SOP-Ad groups, there were no significant changes observed in the biochemical parameters during the experimental period. However, CLP procedure resulted in significant increases in the plasma levels of LDH, ALT, BUN, and creatinine at 24 h (*p* < 0.01 vs. SOP group) **(**Fig. 4a–d). These increased parameters of organ injury were significantly ameliorated by double-dose Ang-(1–7) (*p* < 0.01 vs. CLP group) (Fig. 4a–d), although no significant improvement was found after the administration of single-dose Ang-(1–7).

Following the CLP procedure, the blood glucose level in animals appeared to show a biphasic change: an early elevation at 3 h after CLP and then a late decrease at 24 h after CLP (*p* < 0.001 vs. SOP group) (Fig. 4e). However, the late hypoglycemia was significantly ameliorated by double-dose Ang-(1–7) (*p* = 0.018 vs. CLP group) (Fig. 4e), although no significant improvement was found after the administration of single-dose Ang-(1–7).

### Plasma IL-6 level and tissue superoxide level

**Fig. 5:**
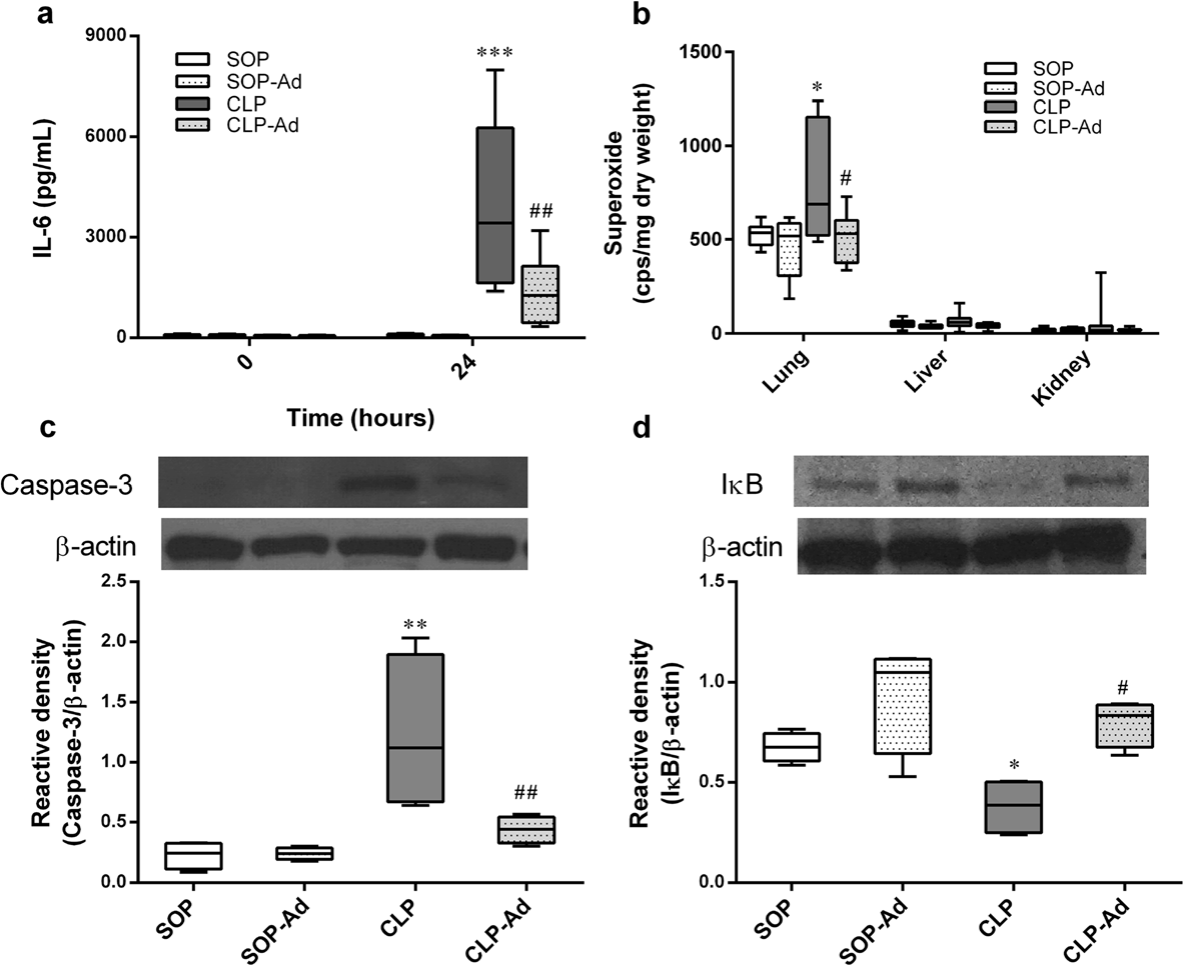
Effects of angiotensin-(1–7) on plasma interleukin-6 and tissue superoxide levels 24 h after procedure. Depicted are the changes in (**a**) plasma interleukin-6 levels and (**b**) tissue superoxide levels in rats that underwent sham operation (SOP, *n* = 5), SOP plus angiotensin-(1–7) administration (1 mg/kg at 3 and 6 h after SOP procedure [SOP-Ad], *n* = 5), cecal ligation and puncture (CLP, *n* = 8), and CLP plus angiotensin-(1–7) administration (same regimen after CLP procedure [CLP-Ad], *n* = 8). Immunoblot analysis of (**c**) cleaved caspase-3 expression and (**d**) the cytoplasmic nuclear factor of kappa light polypeptide gene enhancer in B-cells inhibitor (IκB) in the liver. Rats underwent SOP, SOP plus angiotensin-(1–7) administration (SOP-Ad), CLP, and CLP plus angiotensin-(1–7) administration (CLP-Ad). Liver tissues were harvested at 24 h after surgery. Representative blots are shown on the upper panel, and β-actin served as a loading control. *n* = 4 for each group. Analysis of variance (ANOVA) for repeated measures was used for interleukin-6 analysis, and one-way ANOVA combined with the Newman-Keuls post hoc test was used for the remaining parameters in between-group comparisons. The *p* values for one-way ANOVA for superoxide production, cleaved caspase-3, and IκB expression are 0.02, 0.0035, and 0.0038, respectively. Data are shown as median and IQR. *p < 0.05; **p < 0.01; ***p < 0.001, CLP vs. SOP; ^#^*p* < 0.05; ^##^*p* < 0.01, CLP-Ad vs. CLP

After log transformation, the data of IL-6 and superoxide were normalized and used for statistical analysis. Baseline plasma level of IL-6 did not significantly differ among all groups (Fig. 5a). There were no significant changes observed in the plasma IL-6 level in the SOP and SOP-Ad groups at the end of this study, suggesting neither the sham procedure nor Ang-(1–7) administration could change the plasma level of IL-6. However, CLP led to a significant increase in the plasma level of IL-6 at 24 h (*p* < 0.001 vs. SOP group) (Fig. 5a), which was significantly attenuated by double-dose Ang-(1–7) (*p* = 0.002 vs. CLP group) (Fig. 5a).

A significant increase of superoxide production was observed in lung (*p* = 0.048) but not in liver and kidney tissue homogenates in CLP rats when compared with control rats (Fig. 5b). Ang-(1–7) significantly attenuated the increase of superoxide levels in lung tissues in CLP rats (*p* = 0.039 vs. CLP group) (Fig. 5b).

In the CLP-As group, neither the hemodynamic parameters nor the organ injury indexes showed significant improvement after treatment of CLP rats with single-dose Ang-(1–7). Therefore, oxidative stress, apoptosis, and histological studies were not further performed in this group according to the 3R (reduction, replacement, refinement) spirit.

### Cleaved caspase-3 and IκB expression in liver

To assess the effect of Ang-(1–7) on the apoptosis pathway, cleaved caspase-3 expression was determined. Compared with the SOP and SOP-Ad groups, CLP led to an elevated level of cleaved caspase-3 expression in liver (*p* = 0.009 vs. SOP group), which was significantly alleviated by Ang-(1–7) administration (*p* = 0.018 vs. CLP group) (Fig. 5c). To assess the effect of Ang-(1–7) on the NF-κB signaling pathway, the cytoplasmic IκB expression was determined. Compared with the SOP and SOP-Ad groups, the CLP procedure led to a decreased level of IκB in the cytoplasm (*p* = 0.04 vs. SOP group). However, treatment with Ang-(1–7) significantly inhibited the CLP-induced increase in the cytoplasmic IκB expression in liver (*p* = 0.016 vs. CLP group) (Fig. 5d).

### Histological studies

**Fig. 6:**
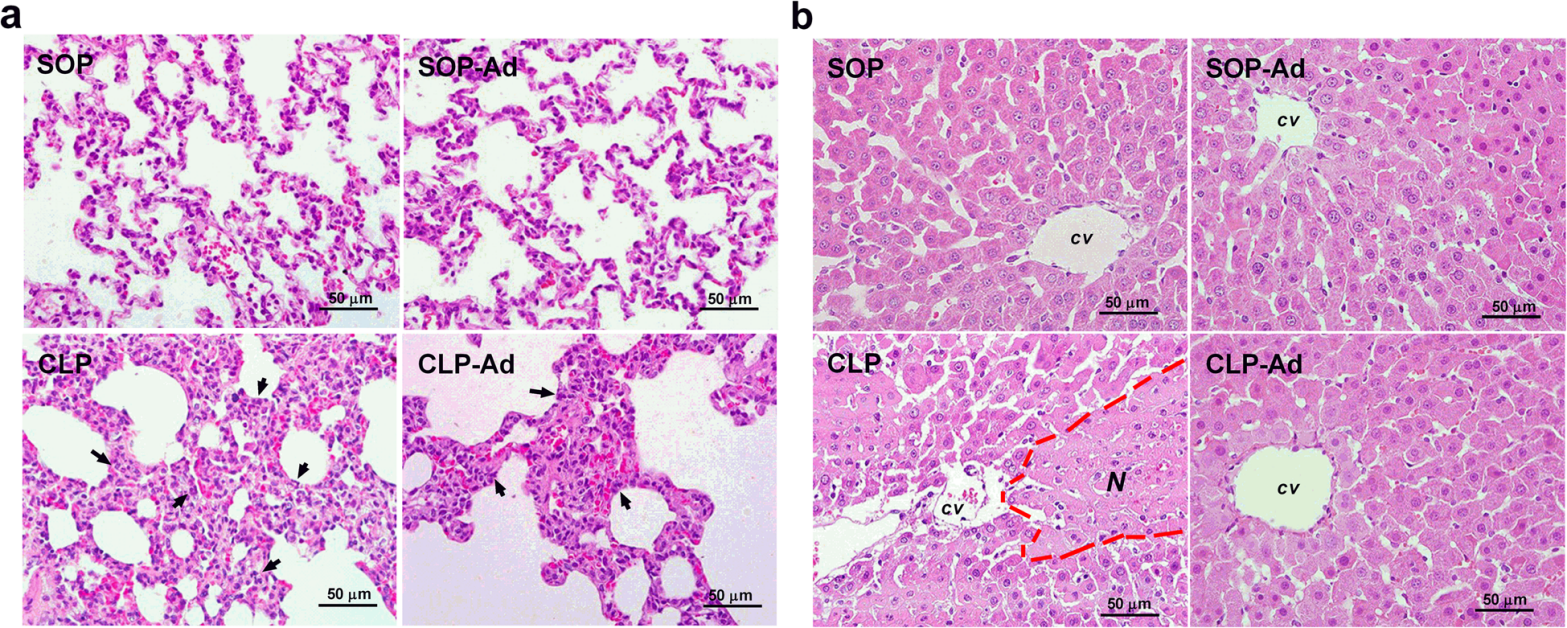
Histopathological studies. Light microscopy of (**a**) lung and (**b**) liver from rats that underwent sham operation (SOP), SOP plus angiotensin-(1–7) administration (1 mg/kg at 3 and 6 h after SOP procedure [SOP-Ad]), cecal ligation and puncture (CLP), and CLP plus angiotensin-(1–7) administration (CLP-Ad). *Arrows* indicate the infiltration of neutrophils. Hematoxylin and eosin staining showed significant necrosis in the liver at 24 h after the CLP procedure. Shown are representative micrographs from three rats per group. Magnification × 400. *N* = Necrotic area, *cv* = Central vein

The lung tissues of the CLP group showed several obvious inflammatory changes, such as lung edema, pulmonary congestion, thickening of the alveolar wall, and areas of inflammatory infiltration at 24 h after the CLP procedure (Fig. 6a). Treatment with Ang-(1–7) seems to alleviate the histopathological changes induced by CLP (Fig. 6a). In addition, the scores were evaluated to determine the thickness of alveolar walls and the increased number of infiltration cells, and the mean pathological score showed no significant difference in CLP rats with Ang-(1–7) treatment compared with CLP rats (1.8 [1.6 to 2] vs. 1 [1 to 1.4], *p* = 0.178 CLP vs. CLP-Ad). However, the liver tissues of CLP group showed necrotic changes, and Ang-(1–7) administration markedly alleviated the histopathological changes induced by CLP (Fig. 6b). The pathological scores of liver necrosis in CLP rats were significantly reduced by treatment of Ang-(1–7) compared with CLP rats (3 [2.5 to 3] vs. 0 [0 to 0], *p* = 0.034 CLP vs. CLP-Ad).

## Discussion

This study demonstrated that the CLP procedure evoked delayed hypotension, hypoglycemia, and multiple organ injuries, characterized by elevated plasma biochemical parameters, histopathological changes, and mortality, which were improved by post-treatment with Ang-(1–7) in a rat model of peritonitis-induced sepsis. Further, Ang-(1–7) significantly attenuated plasma IL-6 production and lung superoxide production, as well as reduced liver IκB and caspase-3 expression, in CLP rats. Thus, these findings suggest, for the first time, to our knowledge, that Ang-(1–7) reduces CLP-induced organ injury, inflammatory response, and even death and may be useful as a therapeutic agent targeting sepsis.

Ang-(1–7), mediated by the Mas receptor, has an anti-inflammatory effect on variable disease processes, such as atherosclerosis, arthritis, and asthma [4]. In an in vitro study, Ang-(1–7) reduced proinflammatory cytokine release in mouse peritoneal macrophages stimulated with LPS [14]. In an in vivo model of endotoxemia, LPS-induced hypothermia, consequent mortality [15], and muscle wasting [22] were attenuated or prevented by Ang-(1–7) through its Mas receptor. CLP is still considered to be the gold standard model of sepsis [23], although this model dose not reproduce completely the complexity of human sepsis. Our present study showed that CLP induced a significant increase of plasma IL-6 production, which was minimized by Ang-(1–7) administration. Moreover, IκB protein, which inactivates the translocation of NF-κB into the nucleus, was enhanced by Ang-(1–7) in CLP rat liver. These results indicate that Ang-(1–7) prevents sepsis-induced inflammation through the IκB/NF-κB pathway.

Increased activity of RAS could lead to oxidative stress and endothelial dysfunction in sepsis [24], which may be associated with the pathogenesis of pulmonary injury [9, 25, 26]. Concerning the counterregulatory actions on RAS, Ang-(1–7) attenuates the Ang II-stimulated increase in lipid peroxidation and decrease in superoxide dismutase activity in mouse heart [27]. In addition, pretreatment with Ang-(1–7) was found to diminish Ang II-induced ROS production in vascular smooth muscle cells [28]. Furthermore, our data showed that the CLP procedure significantly increased superoxide production in lungs, which was attenuated by the post-treatment of rats with Ang-(1–7). There was no significant difference observed in superoxide production in livers and kidney, and it is possible that the CLP procedure induced a trivial effect on these two organs by use of our method.

Recent studies demonstrate that increasing apoptosis in immune cells and parenchymal tissues appeared in the late stage of immunosuppression and consequent organ dysfunction in severe sepsis [16, 29]. Preventing cell apoptosis could improve survival in animal models of severe sepsis [29]. Ang-(1–7) has been reported to inhibit Ang II- and LPS-induced apoptosis in endothelial and epithelial cells [17, 18, 30, 31]. Our study found that Ang-(1–7) ameliorated caspase-3 expression and necrosis changes in CLP rat liver. It indicates that Ang-(1–7) could attenuate apoptosis and cell death induced by severe sepsis. Additionally, apoptosis can be activated by diverse stimuli, including cytokines and ROS [32, 33]. Therefore, Ang-(1–7) may exert its antiapoptotic effect by reducing the production of superoxide and inflammatory cytokines. Taken together, our results suggest that Ang-(1–7) attenuates subsequent organ dysfunction and improves survival in CLP-induced sepsis through its counterregulatory actions to mediate the resolution of inflammation, oxidative stress, and apoptosis.

A previous study showed that pretreatment of 1 mg/kg Ang-(1–7) inhibited LPS-induced cytokine production and hypothermia and thereby protected mice from the fatal consequences of endotoxemia [15]. In the present study, 1 mg/kg Ang-(1–7) was administered at 3 h after CLP procedure (Fig. 1), which did not attenuate organ dysfunction (Figs. 3 and 4). However, this dosage did not affect any hemodynamic and biochemistry parameters in sham control rats during the experimental period. Thereafter, we used post-treatment of 1 mg/kg Ang-(1–7) twice (i.e., at 3 and 6 h after CLP procedure) in the current study.

This study has several limitations. First, the CLP model was performed on young and healthy animals instead of in the clinical setting of elderly patients with comorbidities, although this model is considered the gold standard model of sepsis. Second, we did not use antibiotics in this study to avoid interfering with the effect of Ang-(1–7) on organ function. Third, Ang-(1–7) was administered starting 3 h after the CLP procedure, which does not represent clinical practice in which the therapeutic approach is usually undertaken in the late phase of sepsis. The effect of Ang-(1–7) used in the late phase of sepsis remains to be determined.

## Conclusions

We describe a novel function of Ang-(1–7) in the therapy of CLP-induced sepsis. Ang-(1–7) attenuates multiple organ dysfunction and improves survival in rats with polymicrobial sepsis by reducing the inflammatory response, oxidative stress, and apoptosis characteristics, which may involve IκB/NF-κB signaling. Further studies, and particularly clinical trials, are necessary to clarify the potential adjunct effects of Ang-(1–7) in the early or even late phase of polymicrobial sepsis.

## Abbreviations

ACE2: Angiotensin-converting enzyme 2
ALT: Alanine aminotransferase
Ang: Angiotensin
Ang II: Angiotensin II
BUN: Blood urea nitrogen
CLP: Cecal ligation and puncture
HR: Heart rate
IκB: Nuclear factor of kappa light polypeptide gene enhancer in B-cells inhibitor
IL-6: Interleukin 6
LDH: Lactate dehydrogenase
LPS: Lipopolysaccharide
MAP: Mean arterial pressure
RAS: Renin-angiotensin system
SOP: Sham operation
TBST: Tris-buffered solution containing 0.1% Tween-20
